# Regulatory T Cell Extracellular Vesicles Modify T-Effector Cell Cytokine Production and Protect Against Human Skin Allograft Damage

**DOI:** 10.3389/fcell.2020.00317

**Published:** 2020-05-20

**Authors:** Sim Lai Tung, Giorgia Fanelli, Robert Ian Matthews, Jordan Bazoer, Marilena Letizia, Gema Vizcay-Barrena, Farid N. Faruqu, Christina Philippeos, Rosalind Hannen, Khuloud T. Al-Jamal, Giovanna Lombardi, Lesley Ann Smyth

**Affiliations:** ^1^Immunoregulation Laboratory, MRC Centre for Transplantation, School of Immunology & Microbial Sciences, King’s College London, Guy’s Hospital, London, United Kingdom; ^2^School of Health, Sport and Bioscience, Stratford Campus, University of East London, London, United Kingdom; ^3^Centre for Ultrastructural Imaging, King’s College London, London, United Kingdom; ^4^Institute of Pharmaceutical Science, Faculty of Life Sciences & Medicine, King’s College London, London, United Kingdom; ^5^Centre for Stem Cells & Regenerative Medicine, King’s College London, London, United Kingdom; ^6^Centre for Cell Biology and Cutaneous Research, Blizard Institute, Barts and The London School of Medicine and Dentistry, Queen Mary University of London, London, United Kingdom

**Keywords:** Regulatory T (Treg) cells, extracellular vesicles, cytokines, allograft rejection, miRNA – microRNA

## Abstract

Regulatory T cells (Tregs) are a subpopulation of CD4^+^ T cells with a fundamental role in maintaining immune homeostasis and inhibiting unwanted immune responses using several different mechanisms. Recently, the intercellular transfer of molecules between Tregs and their target cells has been shown via trogocytosis and the release of small extracellular vesicles (sEVs). In this study, CD4^+^CD25^+^CD127^lo^ human Tregs were found to produce sEVs capable of inhibiting the proliferation of effector T cells (Teffs) in a dose dependent manner. These vesicles also modified the cytokine profile of Teffs leading to an increase in the production of IL-4 and IL-10 whilst simultaneously decreasing the levels of IL-6, IL-2, and IFNγ. MicroRNAs found enriched in the Treg EVs were indirectly linked to the changes in the cytokine profile observed. In a humanized mouse skin transplant model, human Treg derived EVs inhibited alloimmune-mediated skin tissue damage by limiting immune cell infiltration. Taken together, Treg sEVs may represent an exciting cell-free therapy to promote transplant survival.

## Introduction

Regulatory T cells (Tregs) are essential for the maintenance of tolerance to self-antigens and immune homeostasis (Shevach, 2018). In humans, a link between the frequency of Tregs within allografts and longevity has been described (Pearce et al., 1993), whilst the adoptive transfer of *ex vivo* expanded murine, or human Tregs, in preclinical murine transplant models significantly prolonged the survival of murine and human skin allografts, respectively (Sagoo et al., 2011; Boardman et al., 2017; Pilat et al., 2019). Given their efficacy *in vivo*, we are currently conducting Phase I/II clinical trials to investigate the therapeutic potential of autologous Treg therapy to promote transplant survival rates in kidney (The ONE Study: NCT02129881) and liver (ThRIL: NCT02166177) transplant recipient patients (Romano et al., 2017; Sánchez-Fueyo et al., 2019). Despite being used in the clinical setting how these cells function and their target cells *in vivo* have yet to be clearly elucidated.

Tregs are a heterogeneous CD4^+^CD25^+^ T cell subpopulation consisting of thymus derived (natural) and peripheral (induced) Tregs which suppress other immune cells, such as effector T cell (Teff) and dendritic cells (DCs) through both cell contact dependent and independent mechanisms (Romano et al., 2019). These include IL-2 deprivation through expression of CD25, production of immune modifying cytokines such as TGFβ, IL-10 and IL-35, induction of target cell death and inhibition of antigen presenting capacity of DCs [reviewed in Shevach (2009)]. Recently, Tregs were found to maintain immune homeostasis through the intercellular acquisition, or trogocytosis, of key components involved in activating Teffs. For example, Samson and colleagues have shown that CTLA-4 expressed on Tregs removed CD80/86 from the surface of antigen presenting cells (APCs), thereby limiting their co-stimulatory capacity (Qureshi et al., 2011). More recently, antigen-specific Tregs that formed strong interactions with peptide pulsed DCs were shown to remove MHC class II: peptide complexes from these cells, reducing their capacity to present antigen (Akkaya et al., 2019). Intercellular communication by Tregs has also been shown to occur via the release of small extracellular vesicles (EVs). CD4^+^CD25^+^ Tregs isolated from rodents [mouse (Smyth et al., 2013; Okoye et al., 2014), and rat (Yu et al., 2013; Aiello et al., 2017)] and humans (Torri et al., 2017; Azimi et al., 2018) were found to produce EVs following TCR activation. These vesicles displayed *in vitro* immune modulatory properties similar to the cell they were derived from [Smyth et al. (2013), Okoye et al. (2014), Torri et al. (2017)]. We have shown that exposure to murine Treg EVs causes (i) a reduction in CD4^+^ Teff cell proliferation as well as IL-2 and IFNγ release (Smyth et al., 2013), and; (ii) an increase in IL-10 production by murine DCs following LPS stimulation (Tung et al., 2018). We attributed these effects to the cell surface immune modulatory molecule CD73, an ecto enzyme involved in adenosine production (Smyth et al., 2013), and specific miRNAs, such as miR-142 and miR-150, present in these vesicles (Tung et al., 2018). Other miRNAs, such as Let-7d and miR-146a-5p have also been linked to the suppressive capacity of these vesicles (Okoye et al., 2014; Torri et al., 2017). Treg-derived EVs have also been shown to transfer iNOS to target cells as a means of disrupting signaling pathways and eliciting a regulatory function (Aiello et al., 2017).

So far, only a few groups have studied the suppressive capacity of Treg EVs *in vivo* in animal models of intestinal inflammation and solid organ transplants. Adoptive transfer of Let-7d deficient murine Tregs into Rag^–/–^ mice reconstituted with CD45RB^hi^ cells failed to prevent intestinal inflammation compared to wild type Tregs (Okoye et al., 2014). The authors demonstrated *in vitro* that this outcome was due to a decreased suppressive activity of Let-7d deficient Treg EVs compared to their untreated counterparts (Okoye et al., 2014). In a rat transplant model, Yu et al. (2013) demonstrated that the administration of Treg vesicles post-transplant prolonged the survival time and function of kidney grafts (Yu et al., 2013). More recently, Aiello et al. (2017) observed that EVs derived from induced Tregs, generated by co-culturing rat CD4^+^CD25^–^ cells with DCs made immature by inhibiting NF-KB, by overexpressing the dominant negative form of IKK2, promoted transplant tolerance only when given in combination with Cyclosporine (Aiello et al., 2017). From these observations, it could be envisaged that like their cell counterpart, human Treg EVs may represent a promising therapeutic tool for treating transplant rejection or autoimmunity.

In this study, we investigated the mechanisms of action of human Treg EVs and their function *in vivo* using an established humanized mouse model of human skin transplantation. Our results demonstrated the immune regulatory role of human Treg EVs *in vivo* and highlighted the possibility of applying them to prevent transplant rejection.

## Materials and Methods

### Human CD4^+^CD25^+^ and CD4^+^CD25^–^ Isolation and Expansion

Anonymous healthy donor blood was obtained from the National Blood Service (NHS Blood and Transplantation, London, United Kingdom) with informed consent and ethical approval (Institutional Review Board of Guy’s Hospital; reference 09/H0707/86). RosetteSep^TM^ (StemCell Technologies, Cambridgeshire, United Kingdom) was used to select CD4^+^ T cells. Subsequently, CD25 Microbeads II (Miltenyi Biotec, Surrey, United Kingdom) were used to separate CD4^+^CD25^+^ (Tregs, Supplementary Figures S1A,B,E) and CD4^+^CD25^–^ (Teffs, Supplementary Figures S1C,D) cells. Tregs were cultured and expanded for 20 days as previously reported (Scottà et al., 2013). Briefly, isolated Tregs were cultured in X-VIVO 15 medium (Lonza, Berkshire, United Kingdom) supplemented with 5% heat-inactivated human AB serum (Biosera, Sussex, United Kingdom) in the presence of anti-CD3/CD28-coated beads (Thermo Fisher Scientific, Paisley, United Kingdom), 100 nM rapamycin (LC-Laboratories, MA, United States) and 1000 U/mL recombinant IL-2 (Proleukin-Novartis, Camberley, Surrey, United Kingdom). Teffs were cultured in the same media in the presence of anti-CD3/CD28-coated beads and 100 U/mL recombinant IL-2. All cell cultures were tested for mycoplasma and all samples used were mycoplasma free.

### EV Purification and Quantitation

Anti-CD3/CD28 beads were removed from cultured cells using a magnet and bead free Tregs and Teffs were cultured in EV-free culture medium for 2 days in the presence of low dose IL-2 (100 IU/ml) in the absence of rapamycin. EV-free media consisted of X-VIVO 15 supplemented with 5% EV free heat-inactivated fetal bovine serum (FBS; Thermo Fisher Scientific). This media was subjected to ultracentrifugation for 18 h at 100,000 × *g* and 4°C using a L8-60M Beckman Coulter ultracentrifuge with a Type 70.1 Ti rotor. To isolate EVs, 5–50 × 10^6^ T cells (2.5 × 10^6^ per ml) were activated using plate-bound anti-CD3 (5 mg/mL, OKT3; Thermo Fisher Scientific) and anti-CD28 (10 mg/mL, αCD28.2) antibodies in EV free media. 24 h post-activation, cell culture media was collected and differential centrifugation preformed. Briefly, culture supernatant was centrifuged at 300 *g* for 10 min, at 2000 *g* for 10 min and passed through a 0.22 μm pore-sized filter (Merck Millipore, MA, United States). EVs were isolated via ultracentrifugation or ExoQuick-TC^TM^ (Systems Biosciences, California, United States). ExoQuick-TC^TM^ was used according to manufacturer’s instructions. Alternatively, EVs were pelleted at 100,000 g using a Beckman L8-60M ultracentrifuge with Beckman Type 70.1 Ti rotor for 1.5 h at 4°C. The EV enriched pellet was washed in PBS at 100,000 g for 1.5 h at 4°C. Ultracentrifugation was employed for transmission electron microscopy, NanoSight LM-10 (NanoSight, Malvern, Kk) and flow cytometry EV analysis. EV enriched pellets were re-suspended in PBS and used immediately except for NanoSight LM-10 analysis where EVs were stored at −80°C. EV isolation via ExoQuick-TC^TM^ was employed for EV RNA extraction, protein analysis, EV suppression assays with subsequent cytokine bead array analysis and for the *in vivo* experiments. EVs pellets derived from ExoQuick-TC^TM^ were re-suspended in EVs-free complete media for *in vitro* analysis and sodium chloride (NaCl) 0.9% w/v saline (B Braun, Sheffield, United Kingdom) for *in vivo* analysis. To measure the concentration and size of the particles isolated, EVs were assessed on a NanoSight LM10 under a constant flow injection. 5 videos of 30 s duration were recorded per sample.

### Electron Microscopy (EM)

10 × 10^6^ Tregs were activated and EVs isolated as described above. EVs were added to Formvar/carbon films with copper grids (TAAB, United Kingdom) and following fixation with 2% glutaraldehyde (Sigma-Aldrich, Dorset, United Kingdom), the grids were washed three times with distilled water. EVs were then stained with 3% uranyl acetate (Sigma-Aldrich) and 2% methylcellulose and analyzed on a transmission electron microscope (FEI Tecnai^TM^ G2 20, Netherlands). All steps were performed at room temperature.

### Cell and EVs Phenotype

All antibodies were purchased from Thermo Fisher Scientific unless otherwise stated and cells were stained using FACS buffer (PBS containing 5 mM EDTA and 1% FCS). Cells were stained with antibodies specific for human; CD4 (OK-T4), CD25 (AE3), CD127 (eBioRDR5), FoxP3 (PCH101), CD39 (eBioA1), CD73 (AD2), CTLA4 (BN13), CD69 (H1.2F4), CD81 (5A6), CD9 (eBioSN4), CD63 (HSC6), TCRαβ (T1089.1A-31) and CCR4 (L291H4). Intracellular staining was performed using a FoxP3 staining buffer set according to manufacturer’s instructions. To stain Treg EVs, EVs were coated onto 4 μm aldehyde/sulfate latex beads (Thermo Fisher Scientific) before the addition of fluorochrome-conjugated antibodies, as previously described (Smyth et al., 2013). Cells and beads were acquired using BD LSR-Fortessa flow cytometer (BD Biosciences, Franklin Lakes, NJ, United States) and analyzed using FlowJo 10 software (Tree Star, OR, United States). CD63 expression on Treg EVs was also assessed using an ExoELISA-ULTRA complete kit (CD63 detection), following manufacture’s instructions (Systems Biosciences).

### SDS Page Gel and Western Blotting

All reagents were purchased from Thermo Fisher Scientific, unless otherwise stated. Treg EVs isolated from (50 × 10^6^) Tregs and Tregs (1 × 10^6^) were lysed in RIPA buffer supplemented with protease inhibitors (Merck, Gillingham, United Kingdom). 10 μgs of denatured protein was run on a 10–20% Novex WedgeWell Protein gel and proteins were transferred onto a PVDF membrane. CD63 and CD81 were assessed using an ExoAb antibody kit following the manufacture’s instructions (Systems Biosciences). Calnexin expression was assessed using a rabbit anti-human/mouse Calnexin primary Ab (Proteintech, Manchester, United Kingdom) followed by a goat anti-rabbit Ig-HRP conjugated secondary Ab (Cell Signaling Technologies MA, United States), both at a 1:1000 dilution. Protein bands were visualized using ECL and a ChemiDoc Imaging System (Bio-Rad, Watford, United Kingdom).

### Mass Spectrometry

Treg EVs, isolated using ExoQuick-TC, were sent for proteomic analysis at Systems Biosciences (SBI Exosome Proteomics Services) and a total EV protein prolife was undertaken. Briefly, according to SBI, EVs were lysed using a sonic probe and 10 μg of sample run on a 10% Bis Tris NuPage gel. An in gel-based digestion, using trypsin, was undertaken to generate peptidic libraries for nano LC-MS/MS analysis using a Waters NanoAcquity HPLC system. Data analysis was undertaken using Scaffold Proteome Software.

### Suppression Assays

1 × 10^5^ CellTrace Violet (CTV, Thermo Fisher Scientific)-labeled non-autologous CD4^+^25^–^ (Teffs) were incubated with or without anti-CD3/CD28 beads (at a 1:40 of bead:Teff ratio). Co-cultures of Tregs:Teffs and Treg EVs:Teffs were set up using varying ratios of cells and EVs, as indicated, for 5 days at 37°C and 5% CO_2_. As a control, the pellet isolated from the same amount of media used to isolate EVs from was used in the suppression assays. CD4^+^ T cell proliferation was assessed by CTV dilution using a BD LSR-Fortessa flow cytometer and analyzed as mentioned previously. Results are shown as percent suppression relative to T cells cultured alone. Culture supernatants were harvested and stored at −20°C for cytokine measurements.

### Cytokine Measurements

Culture supernatants were analyzed using a Human Th1/Th2/Th17 Kit BD^TM^ Cytometric Bead Array (BD Biosciences) according to manufacturer’s protocols. Beads were acquired on a BD LSR-Fortessa flow cytometer and data analyzed using FCAP Array v.3 software. IL-10 was also measured using a human IL-10 ELISA (Thermo Fisher Scientific).

### Animals and Human Skin Xenograft Transplant Model

BALB/c recombination activating gene (RAG)_2_^–/–^γc^–/–^ (BRG) mice were maintained under sterile conditions (Biological Services Unit, New Hunt’s House, King’s College London). All procedures were performed in accordance with all legal, ethical, and institutional requirements (PPL70/7302). Human skin was obtained from routine surgical procedures with informed consent and ethical approval. Skin sections (1.5 cm^2^) were transplanted onto the dorsal region of 10–11 week old BRG mice as previously described (Sagoo et al., 2011; Boardman et al., 2017) and mice received anti-mouse Gr1 antibody (100 ugs; BioXCell, Upper Oxfordshire, United Kingdom) every 4 days via intraperitoneal injection. 6 weeks post-transplant mice received 5 × 10^6^ CD4^+^CD25^–^ cells via intravenous (IV) injection. Control mice received saline. In addition, and concurrently, mice received 1 × 10^6^ Tregs or Treg-derived EVs (derived from 50 × 10^6^ activated Tregs). Control mice received “media EVs” (protein and EVs derived from the EV isolation media) or saline. Transplanted human skin was explanted for histological analysis 5 weeks post-adoptive transfer.

### Histological and Confocal Analysis

Human skin allografts explants were immediately frozen in optimum cutting temperature (OCT) (Thermo Fisher Scientific). Skin sections (8 μm) were fixed with 10% formalin (Thermo Fisher Scientific) before incubating with hematoxylin solution followed by 1% eosin (Merck). For immunofluorescence staining, sections were fixed in 4% paraformaldehyde, and treated with a mixture of 10% goat serum, 0.1% fish skin gelatin, 0.1% Triton X-100 and 0.5% Tween-20 (Merck) in PBS. Next sections were stained with a mixture of the following primary antibodies: anti-human CD3 (polyclonal rabbit; DAKO, Cheshire, United Kingdom) anti-human CD45 (HI30, Thermo Fisher Scientific), anti-human involucrin (CY5, Sigma-Aldrich), anti-human CD31 and anti-human Ki67 (both rabbit polyclonal, Abcam, Cambridge, United Kingdom) for 18 h. Lastly, the sections were stained with secondary antibodies goat anti-mouse Alexa Fluor^®^555 and goat anti-rabbit Alexa Fluor^®^647 antibodies with 4-6-diamidino-2-phenylindole (DAPI) (Thermo Fisher Scientific) and mounted with Fluorescence Mounting Medium (DAKO, Ely, United Kingdom). Nikon C2 + point scanning confocal microscopy (Nikon, Surrey, United Kingdom) and quantitative analysis was performed using ImageJ (FIJI) imaging software.

### MicroRNA Analysis

Total RNA was extracted from Tregs and Treg EVs as previously described (Tung et al., 2018) and a microRNA profile was conducted by Exiqon, United States. The EV miRNA screen was normalized using the global normalization method with RNU6-2 as an endogenous control. For qPCR analysis, cDNA fragments were synthesized using miScript RT Kit (Qiagen) and qPCR reactions were set up with the miScript SYBR Green PCR Kit and primers specific for miR-142-3p and miR-150-5p and RNU6-2, as per manufacturer’s protocols (Qiagen, Hilden, Germany). Samples were run on the Applied Biosystems^TM^ ViiA^TM^ 7 Real time PCR system. Data was analyzed using the ΔΔC_T_ method where RNU6-2 was used as housekeeping controls.

### Statistics

Statistical significance was determined using student’s *t* test, one-way ANOVA with Tukey’s multiple comparisons test. Data was analyzed using PRISM software (GraphPad Software, Inc, California, United States).

## Results

### Human Treg Cells Release Extracellular Vesicles

Several publications from us, and others, have demonstrated that Tregs isolated from mouse (Smyth et al., 2013; Okoye et al., 2014) rat (Yu et al., 2013; Aiello et al., 2017) and human (Torri et al., 2017; Azimi et al., 2018) sources can produce EVs. To characterize human Treg EVs, CD4^+^CD25^+^ human T cells were enriched by magnetic beads separation from healthy donor peripheral blood (average purity of 95.1 ± 3.02%, Supplementary Figures S1A,B), stimulated with anti-CD3/CD28 beads and expanded *ex vivo* in the presence of interleukin-2 (IL-2) and rapamycin, as previously published (Scottà et al., 2013). This protocol has been used for the expansion of Tregs for clinical application in the treatment of renal and liver transplant patients (Romano et al., 2017; Sánchez-Fueyo et al., 2019). The resulting cells maintained a standard Treg phenotype (CD4^+^CD25^hi^CD127^low^) with a high expression of FoxP3, CTLA-4, CD39, and CD73 (Supplementary Figures S2A,B), as previously reported (Scottà et al., 2013; Boardman et al., 2017). Furthermore, these cells also expressed detectable levels of common markers used to define EVs (Théry et al., 2018) including the tetraspanin molecules CD81, CD63, and CD9 (Supplementary Figures S2C,D). As expected, these cells were suppressive, as determined by their ability to inhibit CD4^+^25^–^ T cell proliferation (Supplementary Figure S2E).

**FIGURE 1:**
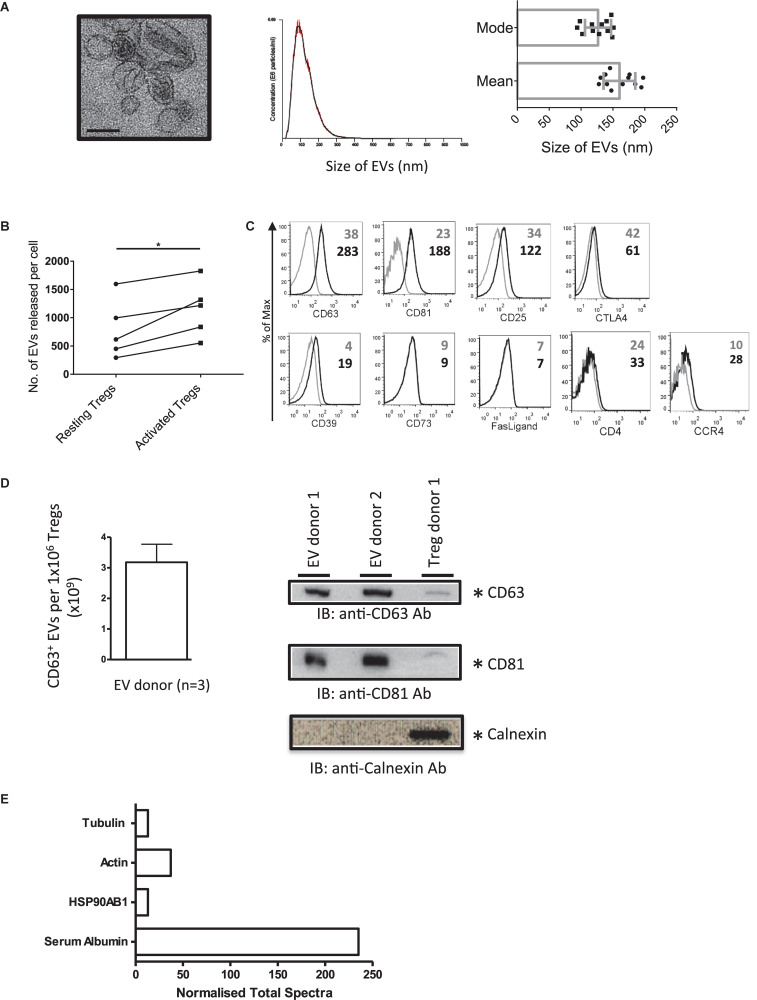
Activated human Tregs release EVs. **(A)** (1st panel) Representative EM image of EVs released by activated human Treg cells. Scale bar indicates 100 nm. 2nd panel, A representative of a size distribution plot for EVs released by TCR activated Tregs. The EV samples were acquired using the NanoSight LM-10 and 5 videos of 30 s duration were recorded. Histogram represents the mean size from all 5 measurements with the red error bars indicating mean ± 1 SEM. 3rd panel, the average mean particle size and the average mode size of Treg EVs isolated from 12 individual donors. **(B)** Graph shows the number of EVs released per cell from resting versus activated Tregs. EVs were isolated from Tregs expanded from 5 donors. **(C)** Representative flow cytometry histogram plots showing the expression of CD63, CD81, CD25, CTLA4, CD39, CD73, Fas-Ligand, CD4, and CCR4 on Treg EVs attached to latex beads following specific antibody staining (black lines). Control samples (gray lines) represent beads stained with the aforementioned antibodies in the absence of EVs. Values indicate the mean fluorescence intensity (MFI) for each of the molecules shown and the control beads. Data represents 1 out of 3 independent experiments. **(D)** Left panel, graph showing the average number of CD63^+^ EVs (×10^9^) ± SEM detected from 1 × 10^6^ Tregs isolated from 3 donors using a CD63 ExoELISA (left panel). Right panel, Western blot showing CD63 (top panel), CD81 (middle panel) and Calnexin (lower panel) expression in Treg EVs lysates (2 individual donors) and Treg cell lysates (1 individual donor). **(E)** Normalized Total Spectra of described proteins found in Treg EVs (1 individual donor).

To isolate EVs, and to avoid the isolation of apoptotic bodies, Tregs were stimulated with plate bound anti-CD3/CD28 antibodies for 24 h and EVs isolated from cell supernatants. The presence of Treg EVs with a cup-shaped morphology was confirmed by electron microscopy (EM) (Figure 1A, left panel). These vesicles had a mean size of 150 nm and a mode of 125 nm, as assessed by NTA suggesting that they are small EVs (sEVs, Figure 1A middle and right panels). Following activation, the number of released sEVs increased significantly with an average of 1.15 × 10^3^ vesicles (range of 0.55 × 10^3^−1.82 × 10^3^ vesicles) being released per activated Treg cell (Figure 1B).

Our previous study showed that murine Treg EVs presented CD63, CD81, CD4, CD25, CTLA-4, and CD73 by flow cytometry using latex beads (Smyth et al., 2013). As such, we next characterized our human Treg EVs phenotypically (Figure 1C). Treg EVs presented CD63 and CD81 as expected, as well as CD25, CD39 molecules and CCR4 (Figure 1C). Low levels of CD4 and CTLA-4 were also observed whilst in contrast, FasL and CD73 were not associated with these vesicles (Figure 1B). The presence of CD63^+^ CD81^+^ on Treg EVs particles was confirmed using a CD63 ExoELISA and Western blotting (Figure 1D).

In accordance with the ISEV position paper on the minimal requirements for reporting EV data (Théry et al., 2018) we assessed several proteins of EV and non-EV origin in our samples. A proteomic screen of the precipitated EV rich pellet, from one donor, was performed. HSP90AB1, actin and tubulin, all previously shown in EVs, were observed (Figure 1E), however, calnexin was not detected (Figure 1D). Previous studies have shown that albumin is a major protein, of non-EV origin, co-isolated with EVs from culture supernatants (Théry et al., 2018). Indeed serum albumin was present in our EV sample (Figure 1E).

Taken together, activated human Tregs released small CD63^+^CD81^+^ vesicles, which express molecules found on their cellular equivalent, including CD25, CD39 as well as the homing receptor CCR4.

### Human Treg EVs Suppress T Cell Proliferation *in vitro*

**FIGURE 2:**
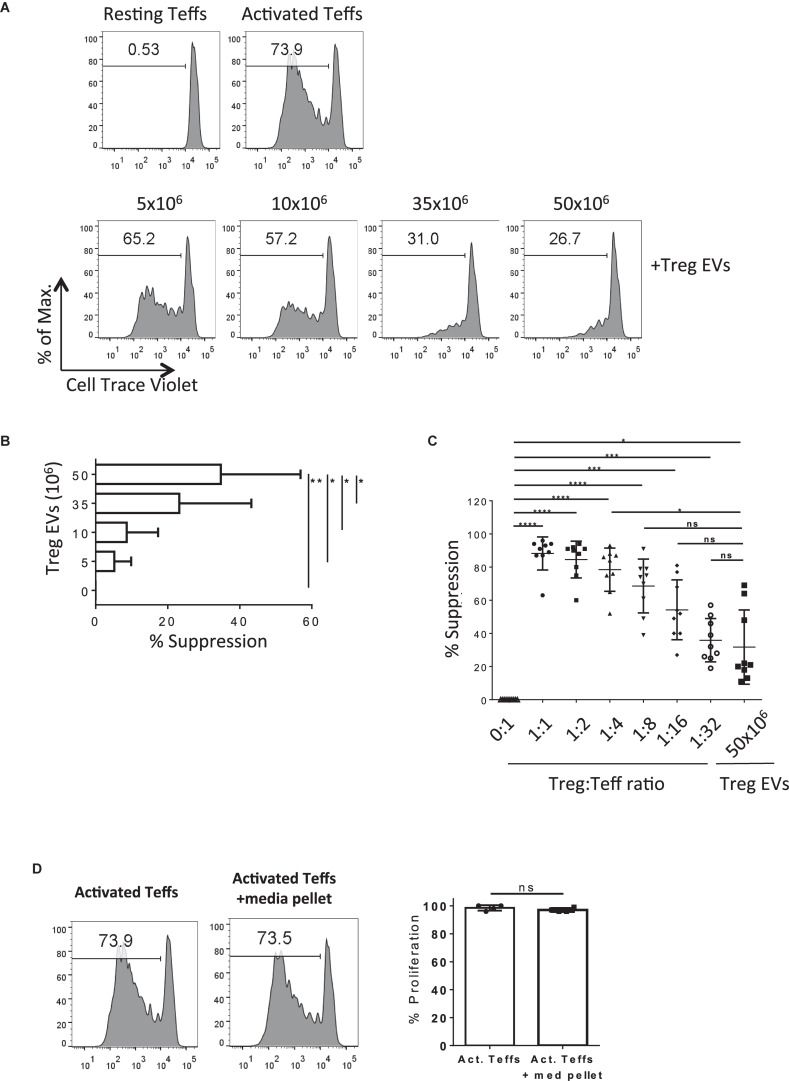
Human Treg EVs are suppressive.**(A)** 1 × 10^5^ Cell Trace Violet (CTV) CD4^+^25^–^ (Teffs) were co-cultured alone (Top left panel, Resting Teffs) or with anti-CD3/CD28 coated beads, in the absence (Top right panel, Activated Teffs) or presence of Treg EVs derived from various numbers of Tregs (Lower panels, + Treg EVs). After 5 days the proliferation of the CTV Teffs was assessed by flow cytometry. Representative data of the percentage proliferation from an individual donor is shown. **(B)** Average% suppression of Teff proliferation following exposure to increasing dose of Treg EVs. Data is from 3 independent experiments using 3 individual donors. Bars show mean + SEM. **(C)** Graph represents the% suppression of Teffs from at least 7–9 independent donors, each shown as an individual point, following co-culture with various ratios of Treg cells or Treg EVs derived from 50 × 10^6^ Treg cells. The mean ± SEM is shown. In all panels data are expressed as percentage of suppression of Teff proliferation relative to Teffs cultured alone with anti-CD3/28 beads. **(D)** Teffs were activated with anti-CD3/28 beads for 5 days in the absence (Activated Teffs) or presence of proteins and EVs isolated from culture media (Activated Teffs + media pellet). Flow cytometry histogram plots represent the proliferation of Teffs as measured by CTV dilution. Data shown represents one of 6 individual experiments with the pooled percentage CFSE^–/lo^ cells (% proliferation) shown in the right panel. Statistical significance was tested using one-way ANOVA where *p < 0.05, **p < 0.01, ***p < 0.001, ****p < 0.0001, and ns, non-significant.

Next, we tested the suppressive capacity of human CD4^+^CD25^+^ T cell derived EVs *in vitro*. Cell Trace Violet^®^ (CTV)-labeled CD4^+^CD25^–^ Teffs were stimulated with anti-CD3/CD28 coated beads in the presence or absence of different quantities of Treg EVs (Figures 2A). As expected, Teff proliferation was suppressed in the presence of Treg EVs in a dose-dependent manner with the maximal inhibition of T cell proliferation observed in the presence of Treg EVs isolated from 50 × 10^6^ cells (Figures 2A). This was constant across several different donor Treg EVs (Figure 2B, 5 × 10^6^ Treg-derived EVs versus 50 × 10^6^ Treg-derived EVs *p* = 0.0438; 10 × 10^6^ Treg-derived EVs versus 50 × 10^6^ Treg-derived EVs *p* = 0.0317; 35 × 10^6^ Treg-derived EVs versus 50 × 10^6^ Treg-derived EVs *p* = 0.0190).

Next, the efficiency of Treg EVs to suppress T cell proliferation compared with Tregs was addressed. The suppression of T cell responses observed in the presence of 50 × 10^6^ Treg-derived EVs was equivalent to those observed when the Teff:Treg ratio was at 1:8 (Figure 2C), suggesting that whilst Treg EVs are immune modulatory, they are less efficient at inducing suppression than the Treg cells themselves in the *in vitro* assay used. Given that the protocol used to isolate the Treg EVs co-precipitated albumin the contribution of this, and other non-EV proteins, to the above observations was assessed. To do this a Treg EV free control; “pellet” isolated from similar volumes of media used to culture cells during activation, was included in the assays. Importantly, no inhibition of Teff proliferation was observed in the absence of Treg EVs (Figures 2D). Taken together, our observations highlight the capacity of Treg-derived EVs to inhibit Teff proliferation *in vitro*.

### Human Treg EVs Modulate Teffs Cytokine Production

**FIGURE 3:**
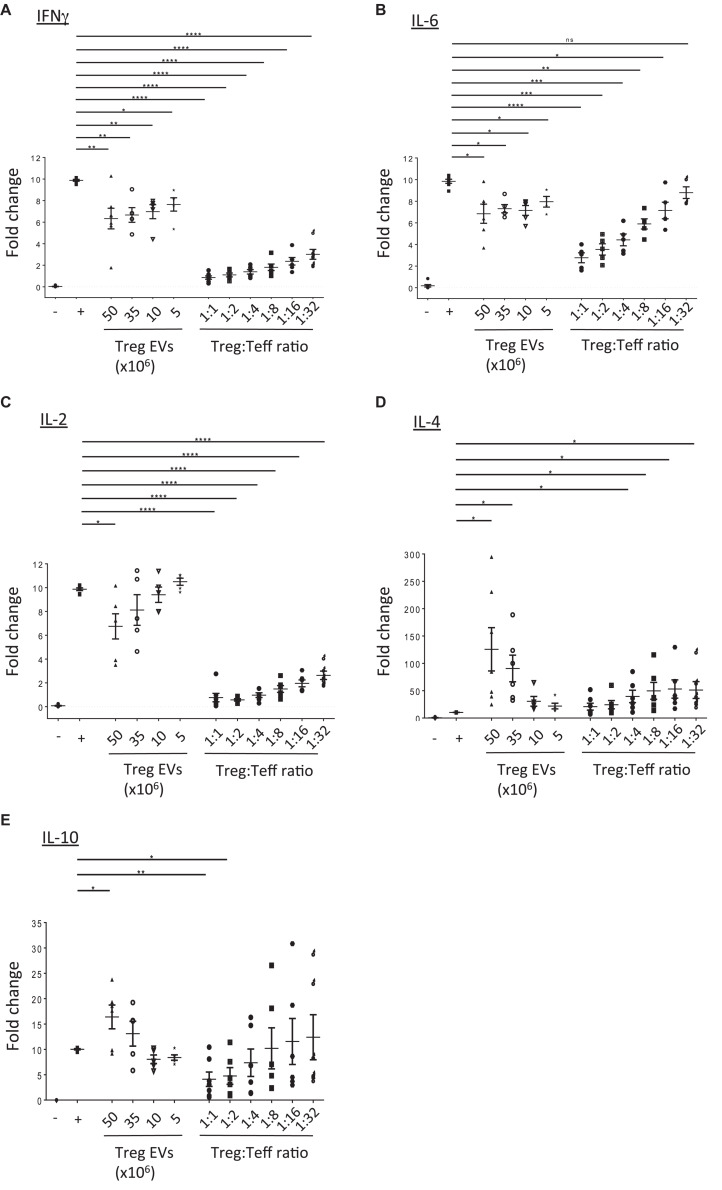
Treg EVs modify cytokine production. CD4^+^25^–^ (Teffs) cells were co-cultured alone (−) or with anti-CD3/CD28 coated beads in the absence (+) or presence of Treg EVs derived from various numbers of Tregs (Treg EVs × 10^6^) or different ratios of Tregs. After 5 days the presence of **(A)** IFNγ, **(B)** IL-6, **(C)** IL-2, **(D)** IL-4, and **(E)** IL-10 was assessed by flow cytometry. Individual graphs **(A–E)** show the relevant cytokine levels produced by Teffs exposed to Tregs or Treg EVs isolated from 7 to 9 individual donors. Each assay was performed as a technical triplicate. Data is shown as the fold change relative to activated Teffs (+), which was set arbitrarily as 10. Statistical significance was tested using one-way ANOVA; mean ± SEM is shown where *p < 0.05, **p < 0.01, ***p < 0.001, ****p < 0.0001, and ns, non-significant.

Next, the direct modulation of Teff function by human Treg EVs was evaluated. Teffs were stimulated with anti-CD3/CD28 beads in the presence or absence of either Treg cells or Treg EVs and cytokine production was evaluated. Teffs produced high levels of IFNγ, IL-2, and IL-6, which were significantly reduced in the presence of Treg cells in a dose dependent manner. Exposure to Treg EVs significantly reduced IFNγ and IL-6 secretion in a dose dependent manner (Figures 3A,B) while IL-2 was significantly reduced following exposure to 50 × 10^6^ Treg derived EVs only (Figure 3C). Like the proliferation data, the reduction of these pro-inflammatory cytokines by Treg EVs was less efficient than those induced by their cell counterparts.

In contrast, we observed a significant increase in IL-4 and IL-10 cytokine production in the supernatants of Teffs following co-culture with high doses of Treg derived EVs (Figures 3D,E, respectively). Interestingly, the increased level of cytokines observed were greater than those produced in the presence of Tregs (1:1 and 1:2 Teff:Treg ratio). This increase in IL-10 was not due to this cytokine being present in the Treg EVs themselves. No IL-10 was detected in lysed EVs using an IL-10 specific ELISA (data not shown).

**FIGURE 4:**
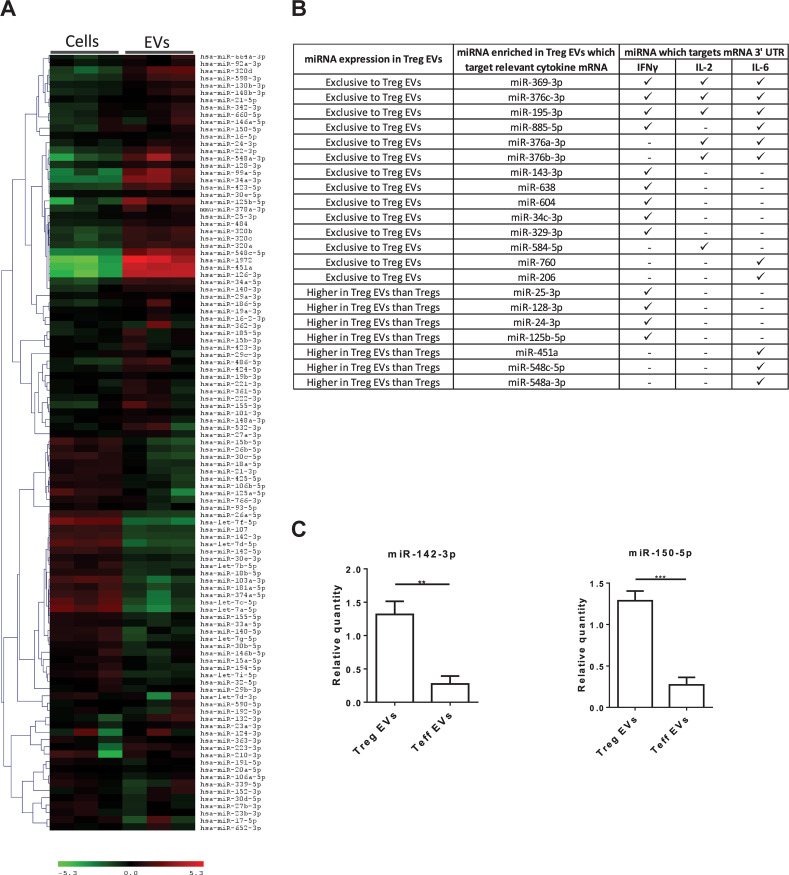
MiRNA present in Treg EVs target the 3′ UTR of mRNA of IL-2, IL-6 and IFNγ cytokines. **(A)** Microarray heat map diagram showing a two-way unsupervised hierarchical clustering of miRNAs by Tregs and Treg EV miRNAs. Cells and EVs from three independent donors are shown. **(B)** Table of miRNAs found exclusively in Treg EVs or enriched in Treg EVs compared to Tregs and their targeting prediction to 3′ UTR of IFNγ, IL-2 and/or IL-6 mRNA. **(C)**. Bar graphs (mean ± SEM) showing the relative quantity (RQ) of miR-142-3p and miR-150-5p expression in Tregs EVs and control EVs, as measured by qPCR and normalized relative to RNU6-2. Data was pooled from 3 individual experiments that were performed in triplicates. Statistical significance was determined using an One way ANOVA and Tukey’s multiple comparison test, where **p < 0.01 and ***p < 0.001.

Murine Treg EVs contain miRNAs which, when acquired, modify the cytokine profile of the target cells (Tung et al., 2018). To assess whether miRNAs present in our human Treg EVs could potentially affect pro-inflammatory cytokine production, a microarray screen was undertaken (Figure 4A). A bioinformatics tool (microRNA.org) was used to predict whether miRNAs highly expressed in Treg EVs, as compared to the Tregs, targeted the 3′UTRs of IFNγ, IL-2 and IL-6 mRNA (Supplementary Figures S3A–C). As shown in Figure 4B, several miRNAs were identified which were predicted to recognize one or more target sequences within each of the aforementioned pro-inflammatory cytokine mRNAs. In addition, two miRNAs present in murine Treg EVs, miR-142 and miR-150, linked with reduced IL-6 (Sun et al., 2013) and IL-10 production in DCs and T cells, respectively (King et al., 2016), were also identified and their expression validated by qPCR (Figure 4C).

Overall, these results demonstrate that like their cell counterparts, human Treg EVs have the capacity to skew CD4^+^ T cells toward an anti-inflammatory state, with Treg EVs being more efficient at doing so than Tregs. We suggest that the miRNAs enriched in the human Treg EVs maybe responsible for the modulation of the cytokine profile observed, although this requires validation. Given these findings we next tested whether the protective effects seen *in vitro* could be translated *in vivo*.

### Human Treg EVs Function *in vivo*

To investigate the *in vivo* effect of human Treg EVs, a well-established humanized mouse model of skin transplantation was selected (Sagoo et al., 2011; Putnam et al., 2013; Boardman et al., 2017). In this model, human skin grafts were transplanted onto immune deficient BALB/c Rag_2_^–/–^γc^–/–^ mice prior to reconstituted with allogeneic human CD4^+^CD25^–^ T cells alone or in combination with autologous Tregs or Treg EVs (Supplementary Figure S4A). H + E staining of human skin isolated from mice 30 days after being treated with CD4^+^CD25^–^ T cells displayed an irregular stratum corneum, disrupted epidermis and dense cellular infiltration compared to saline-treated control mice (Supplementary Figure S4B). Additionally, the epidermal thickness and the rete ridge height were significantly increased (Supplementary Figures S4C,D), indicating an alloimmune-mediated inflammatory response (Sagoo et al., 2011; Putnam et al., 2013; Boardman et al., 2017). Conversely, the injection of CD4^+^CD25^–^ T cells in the presence of Treg EVs resulted in a smooth stratum corneum as well as a significant decrease in epidermis thickness, rete ridge height (*p* < 0.05) and cellular infiltration (Supplementary Figures S4C,D).

**FIGURE 5:**
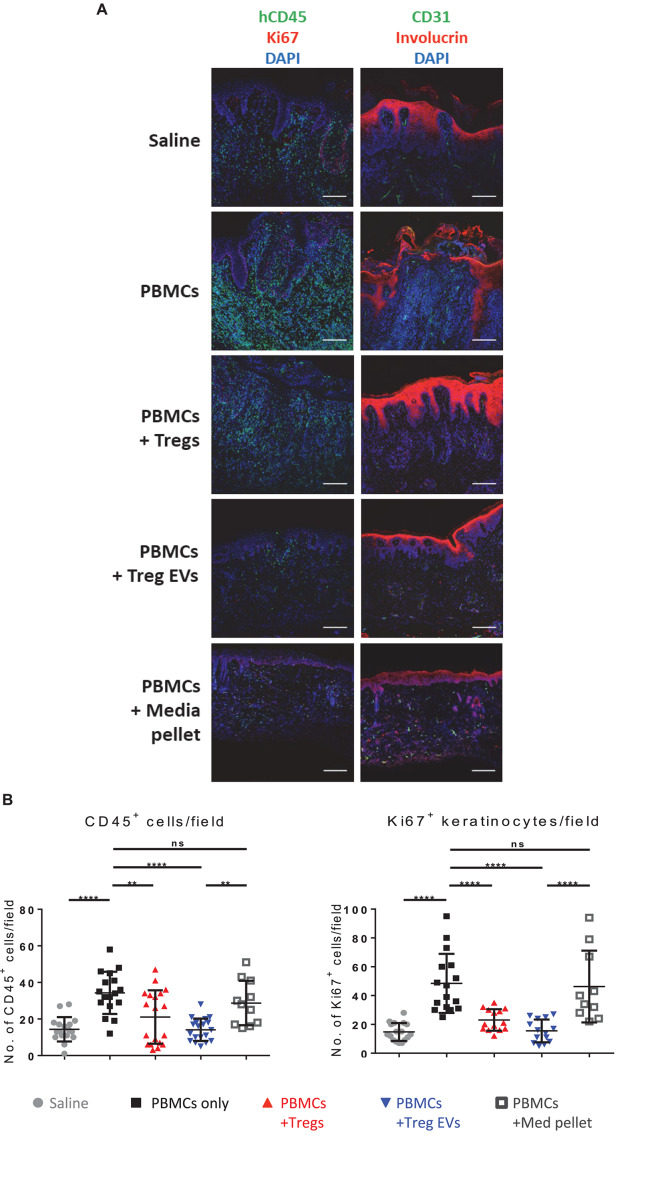
Human Treg EVs protect against alloimmune mediated human skin allograft damage. BALB/c Rag2^–/–^γc^–/–^ mice were transplanted with human skin and 6 weeks later mice were reconstituted with 5 × 10^6^ allogenic CD4^+^25^–^ T cells plus polyclonal 1 × 10^6^ Tregs or Treg EVs derived from 50 × 10^6^ Tregs. Control mice received saline only or control EVs (media EVs). Human skin grafts were removed 5 weeks post-injection, and cryopreserved sections were fixed and stained for human CD45/Ki67/DAPI **(A)**, or CD31/involucrin/DAPI **(A)**. Representative images of human skin graft sections with the various treatments are shown. **(B)** Quantification of the number of human CD45^+^ cells and Ki67^+^ keratinocytes cells per field of view was performed using NIS Elements and FIJI imaging software. Results represent 3–9 mice per group where four to six fields of view were quantified per section and data are representative of 5 individual experiments. Statistical significance was tested using one-way ANOVA and Turkey multiple comparison *post hoc* test where *p < 0.05, **p < 0.01, ***p < 0.001, ****p < 0.0001, and ns, non-significant. DAPI, 4’,6-diamidino-2-phenylindole.

To validate these findings, the expression of specific immune cell and skin-related markers was assessed by three-color immunofluorescence microscopy. As expected, compared to saline controls, grafts from CD4^+^CD25^–^ T cells treated mice showed active inflammation with human CD3^+^ and CD45^+^ cell infiltrates (Figures 5A,B and Supplementary Figure S5) and loss of dermo-epidermal integrity, mediated by allogeneic human leucocytes (Figure 5A). Quantitative analysis of histology findings demonstrated that treatment with Treg EVs, but not proteins isolated in the media pellet, significantly decreased the number of infiltrating human CD45^+^ cells (Figure 5B, left panel), which correlated with significantly fewer CD3^+^ T cells (Supplementary Figures 5A,B). In addition, Treg EV treatment significantly decreased the frequency of Ki67^+^ proliferating keratinocytes (Figure 5B, right panel). All these parameters were reduced in skin transplants following Treg treatment, as previously shown (Putnam et al., 2013; Boardman et al., 2017). These results highlight the efficacy of Treg EVs in protecting allografts *in vivo* and suggest that these vesicles have relevance in clinical application to prevent graft rejection.

## Discussion

In this study, we show that TCR-activation of human Tregs results in the release of small EVs capable of suppressing T cell proliferation and altering their cytokine profile in favor of IL-4 and IL-10 secretion. In a humanized mouse model, human Treg-derived EVs protected human skin grafts from alloimmune-mediated damage. Taken together, these observations suggest that human Treg EVs are immune regulators, which may represent a translatable therapy for the treatment of transplant rejection.

Like murine Tregs, EVs released by their human counterparts inhibit T cell proliferation in a dose dependent manner (Smyth et al., 2013). The exact mechanism behind this observation is unknown, however, one possible explanation is based on the evidence that Treg EVs express Treg-associated markers such as CD39 and CD25. Given that both of these molecules have been linked to the immunosuppressive nature of Tregs, it is tempting to suggest that CD25 and CD39 expression on Treg EVs may explain the inhibition of proliferation observed. The presence of CD25, a high affinity receptor for IL-2, on murine Tregs and T cell derived EVs has previously been reported (Nolte-t Hoen et al., 2004; Smyth et al., 2013). It is tempting to speculate that CD25^+^ Treg derived EVs bind to the cell surface of T cells, leading to depletion of IL-2 from the microenvironment and apoptosis of Teffs. IL-2 was indeed reduced in the presence of high concentrations of Treg EVs, suggesting that CD25 on Treg EVs could be playing a similar role, driving the immunosuppressive response, although this needs to be validated.

Ectoenzymes CD39 and CD73 present on Tregs work together to convert pro-inflammatory ATP into anti-inflammatory adenosine (Deaglio et al., 2007), which can be taken up by adenosine A2 receptors to suppress target cell function and provide an anti-inflammatory microenvironment. CD73 expression contributed directly to the suppressive nature of mouse Treg EVs (Smyth et al., 2013), however, a similar possibility does not exist with the human counterparts, as the human derived EVs do not express this molecule. Although our human Treg EVs only contained CD39, as human T cells express A2 adenosine receptors (Koshiba et al., 1999; Ohta et al., 2009; Linden and Cekic, 2012) it is possible that the combination of these proteins on Teffs following Treg EV fusion resulted in the immune suppression observed in our co-cultures.

Another possible explanation, although not mutually exclusive, is the function of miRNAs identified within the EVs. Recently, Torri et al. highlighted that human Treg EVs were highly enriched in miR-146a-5p, which suppressed proliferation of CD4^+^ T cells by inhibiting Stat1 and Irak2 mRNA levels (Torri et al., 2017). Like this study, we also observed miR-146a-5p in Treg EVs, suggesting that this miRNA may contribute to the reduced Teff proliferation observed.

In our study, we observed a modulation of cytokines produced by T cells following exposure to Treg EVs. Treg EVs reduced IL-6 whilst increasing IL-4 and IL-10 production. IL-6 is known to work in concert with TGFβ to cause degradation of FOXP3 (Gao et al., 2012) and promote the differentiation of pro-inflammatory Th17 cells from naïve CD4^+^ T cells (Bettelli et al., 2006). In contrast, in the absence of IL-6, naïve T cells are more prone to commit toward a Treg compartment (Bettelli et al., 2006). As such, by reducing the presence of detectable IL-6, it is possible that Treg derived EVs both hinder the differentiation of Th17 cells and promote the development of immunosuppressive Tregs. Furthermore, IL-4 is a critical factor involved in skewing naïve T cell differentiation toward a Th2 phenotype.

It is known that EVs are carriers of cytokines (Clayton et al., 2007; Szabó et al., 2014; Konadu et al., 2015; Tokarz et al., 2015) it is therefore conceivable, and another way that EVs modulate the immune response, that the presence of cytokines such as IL-4 and IL-10 are found within the human Treg EVs themselves. However, we have previously shown that murine Treg EVs did not contain IL-10 (Tung et al., 2018) and confirmed this finding with human Treg EVs. Like our current study, we previously observed an increase of IL-10 production in LPS activated BMDCs following treatment with murine Treg EVs, suggesting that something intrinsic to the Treg EVs increased IL-10, and presumably IL-4, production in recipient cells. Several components of the Treg EVs maybe responsible for this including miRNAs. We have recently suggested that miR-150 expressed in murine Treg EVs altered cytokine production in LPS activated DCs leading to an increase in IL-10 (Tung et al., 2018). Our conclusion was based on the published findings of King et al. (2016) who observed that miR-150 was overexpressed in IL-10 secreting CD46-induced human CD4^+^ T cells (King et al., 2016). The same authors demonstrated that inhibiting miR-150, using a miR-150 antisense locked nucleic acid (LNA), led to a decrease in IL-10 production by these cells (King et al., 2016). In this study, we have shown that miR-150 is present in human Treg EVs, suggesting that it might be responsible for the modulation of the IL-10 cytokine profile observed.

As mentioned above, IL-6 is a pro-inflammatory cytokine, produced by many cells including T cells, which has a pleotropic effect on cells of the innate and adaptive immune system (Tanaka et al., 2014). This cytokine has also been shown to drive transplant rejection with Zhao et al. (2012) demonstrating that, with co-stimulatory blockade (CTLA-4-Ig), wild-type C57BL/6 mice rejected fully mismatched BALB/c cardiac grafts but IL-6-deficient C57BL/6 mice accepted these allografts. The authors suggested that blocking IL-6 production and IL-6 signaling in combination with co-stimulatory blockade inhibited Th1 responses, and promoted transplant tolerance (Zhao et al., 2012). Recently, the role of IL-6 in allograft rejection was revisited (Pilat et al., 2019). These authors showed that in combination with rapamycin and an anti-IL-2 monoclonal antibody plus IL-2-complex, blocking IL-6 prolonged MHC mismatched skin transplants for greater than 75 days (Pilat et al., 2019). Here, we show that T cell derived IL-6 production was inhibited in the presence of Treg EVs, suggesting one way that these vesicles may have abrogate T cell alloresponses seen in our mouse model. In addition, early work by Bushell et al. (1999) showed that IL-4 plays a role in tolerance induction to an alloantigen following Treg transfer (Bushell et al., 1999). Therefore increasing IL-4 levels through administration of Treg EVs maybe beneficial for transplant outcomes. Our data highlight that these vesicles could play a protective role in the setting of transplantation. The novel finding that human Tregs EVs prevented alloimmune-mediated damage in human skin allografts, following administration, highlights their potential as a novel cell-free therapy for inhibiting inflammation. The inhibition of IL-6, together with the modulation of IL-4, further underlines the potential of Treg EVs as immunotherapy reagents for transplant patients.

In summary, human Treg EVs inhibit T cell proliferation and cytokine production *in vitro* and protect human skin grafts from alloimmune-mediated damage *in vivo*. Given the latter, and as we previously suggested (Agarwal et al., 2014), these vesicles have the potential to be translated into the clinic as a stand-alone personalized therapy. One major development, that will help the translation of Treg EVs into the clinic, is the recent publication of data from a phase 1 clinical trial on the efficacy of autologous human Tregs in a liver transplant setting (Sánchez-Fueyo et al., 2019). These authors highlighted that administration of these cells to patients post-transplant was safe and led to a transient increased in Treg numbers whilst decreasing CD8^+^ donor-specific responses over time following injection, hence demonstrating efficacy. These important findings align with our speculation that Treg EVs may have a similar effect to their cellular counterparts *in vivo*. Given that these authors found no adverse affects with Tregs helps pave the way for their EVs to be used in a similar setting. Whether these vesicles are best used in combination with Tregs cells has yet to be addressed.

In our previous review we discussed several hurdles that required addressing before human Treg EVs could be used in a clinical setting. These included designing protocols for optimizing *ex vivo* isolation of GMP-grade Treg EVs, improving scalability and assessing reproducibility, a full assessment of protein contaminants, identification of *in vivo* targets as well as working out a dosing regime. Although many of these are still relevant we feel that our protocol of EV isolation allows reproducibility, as determined by the consistency in EV miRNAs expression across several donors. Since the publication of our review several key advances such as GMP compatible EV isolation reagents, bioreactors to allow scalability and tangential flow filtration (Busatto et al., 2018) are transforming EVs research, with several companies now attempting to create off the shelf EV products for patient use. Given this, and the fact that DC-derived EVs have been used in phase II clinical trials for the treatment of non-small cell lung cancer, and were found to have efficacy (Besse et al., 2016, achieving a similar outcome for Treg EVs within liver or kidney transplantation maybe feasible.

## Data Availability Statement

The datasets generated for this study are available on request to the corresponding author.

## Ethics Statement

For human PBMCs used *in vitro* and *in vivo* experiments, healthy volunteers gave written formal consent. Human skin was obtained from routine surgical procedures with informed consent and ethical approval (Guy’s and St. Thomas’ NHS Foundation Trust and King’s College London; reference 06/Q0704/18). Animal protocols were approved and conducted in accredited facilities in accordance with The Home Office United Kingdom Animals (Scientific Procedures) Act 1986 (Home Office license number PPL 70/7302).

## Author Contributions

ST conceived, designed and conducted experiments, analyzed data and wrote and edited the manuscript. GF, RM, ML, CP, and JB conducted experiments and analyzed data. GV-B performed the electron microscope studies. FF and KA-J provided assistance and interpreted data from the NanoSight analysis and provided reagents. RH provided human skin samples. GL designed and critically reviewed and edited the manuscript. LS secured funding, conceived, designed and conducted experiments and wrote and edited the manuscript.

## Conflict of Interest

The authors declare that the research was conducted in the absence of any commercial or financial relationships that could be construed as a potential conflict of interest.

## Abbreviations

Tregs: Regulatory T cells (Tregs)
EVs: Extracellular vesicles (EVs)
DCs: Effector T cell (Teff) and Dendritic cells (DCs).
