# Supplementary material for: Regulatory T Cell Extracellular Vesicles Modify T-Effector Cell Cytokine Production and Protect Against Human Skin Allograft Damage
Source: Front Cell Dev Biol. 2020 May 20;8:317. doi: 10.3389/fcell.2020.00317 (PMC7251034; doi:10.3389/fcell.2020.00317)
Supplement: Supplementary file 1 [file Data_Sheet_1.PDF]

Supplementary Materials:

Figure S1.

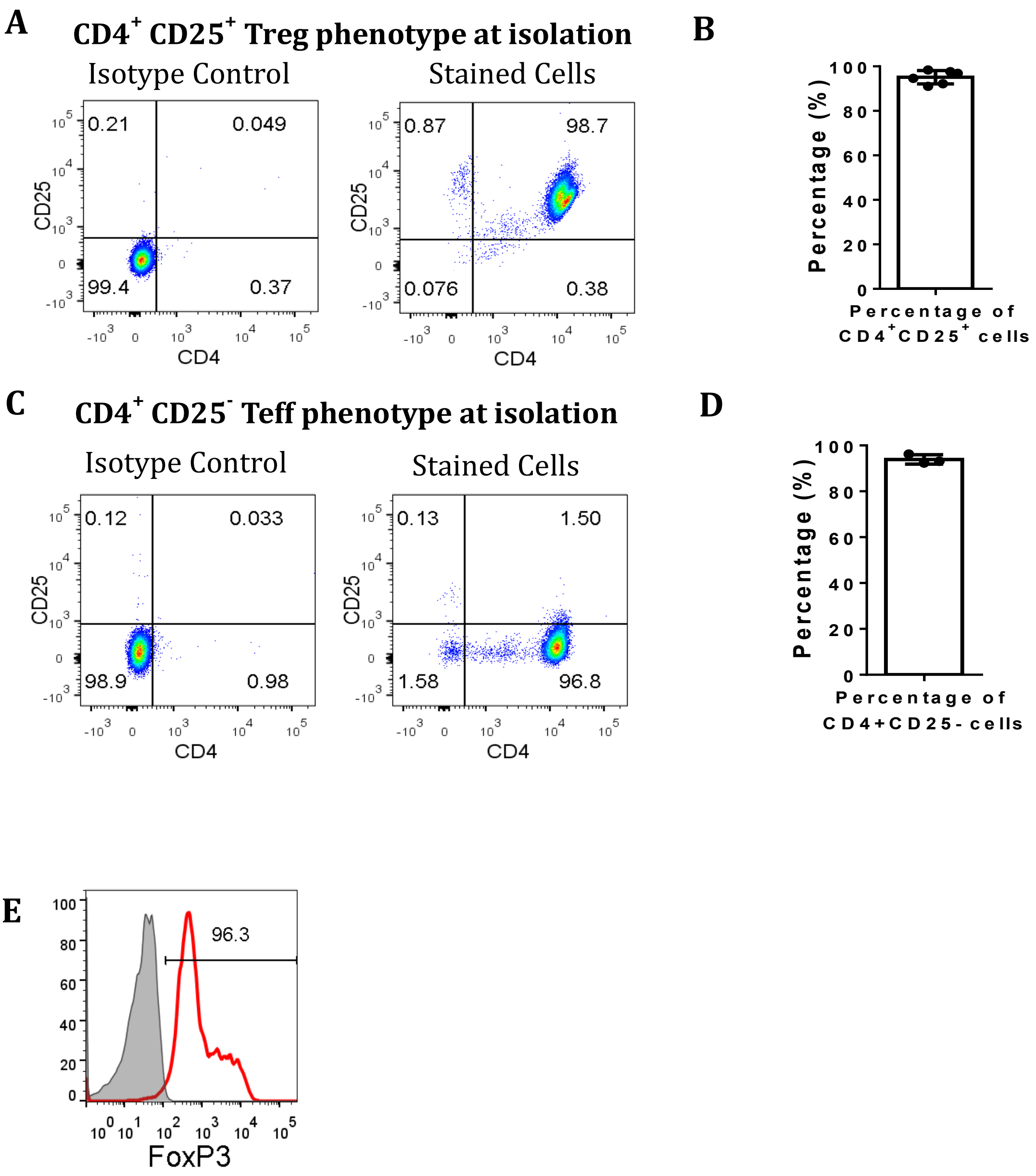

Fig. S1. Purity of both human CD4$^+$CD25$^+$ and CD4$^+$CD25$^-$ T cells after isolation. CD4$^+$CD25$^+$ and CD4$^+$CD25$^-$ T cells were isolated from peripheral blood using magnetic bead isolation. **(A and C)** Flow cytometry plots representing the cell purity following isolation as assessed by the expression of CD4 and CD25 (stained cells) or

controls (isotypes controls). **(B and D)** Data representing the mean percentage of $CD4^{+}CD25^{+}$ cells or $CD4^{+}CD25^{-}$ T cells +/-SEM pooled from 6 donors. (E) Expression of FoxP3 (red line) on freshly isolated $CD4^{+}CD25^{+}$ T cells. Grey bar is the isotype control.

Figure S2.

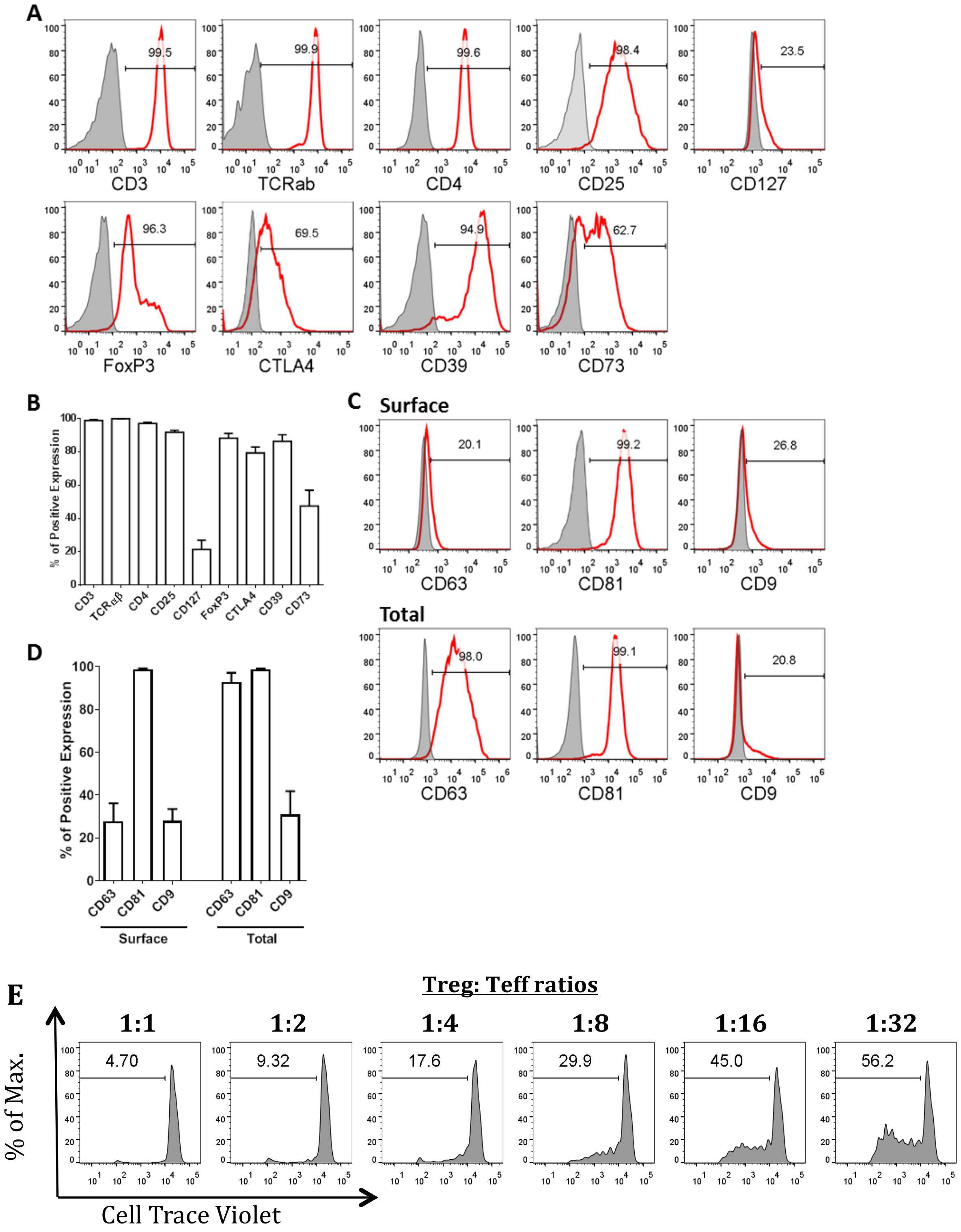

Fig. S2. Phenotype and function of *ex vivo* expanded Tregs. CD4^{+}CD25^{+} T cells were expanded with anti-CD3/CD28 beads in the presence of rapamycin and IL-2. **(A-B)**

Flow cytometry histogram plots representing the expression of CD3, TCRαβ, CD4, CD25, CD127, FoxP3, CTLA-4, CD39 and CD73 (red histogram) on the expanded cells as compared to isotype controls (grey histograms). Data representing the mean percentage expression +/-SEM, of the individual markers shown, from flow data pooled from 3-12 individual donors. **(C-D)** Flow cytometry plots representing the surface and total expression of CD63, CD81 and CD9 on $CD4^{+}CD25^{+}$ rapamycin and IL-2 expanded cells. Data represent the mean percentage expression of surface or total CD63, CD81 and CD9 +/- SEM pooled from 3 individual donors. **(E)** Polyclonal suppression assay; Tregs and autologous Teffs were co-cultured at various ratios in the presence of anti-CD3/28 beads for 5 days. Flow cytometry histogram plots represent the proliferation of Teffs as measured by CellTrace Violet dilution. Data shown represents one of 12 individual experiments.

Figure S3.

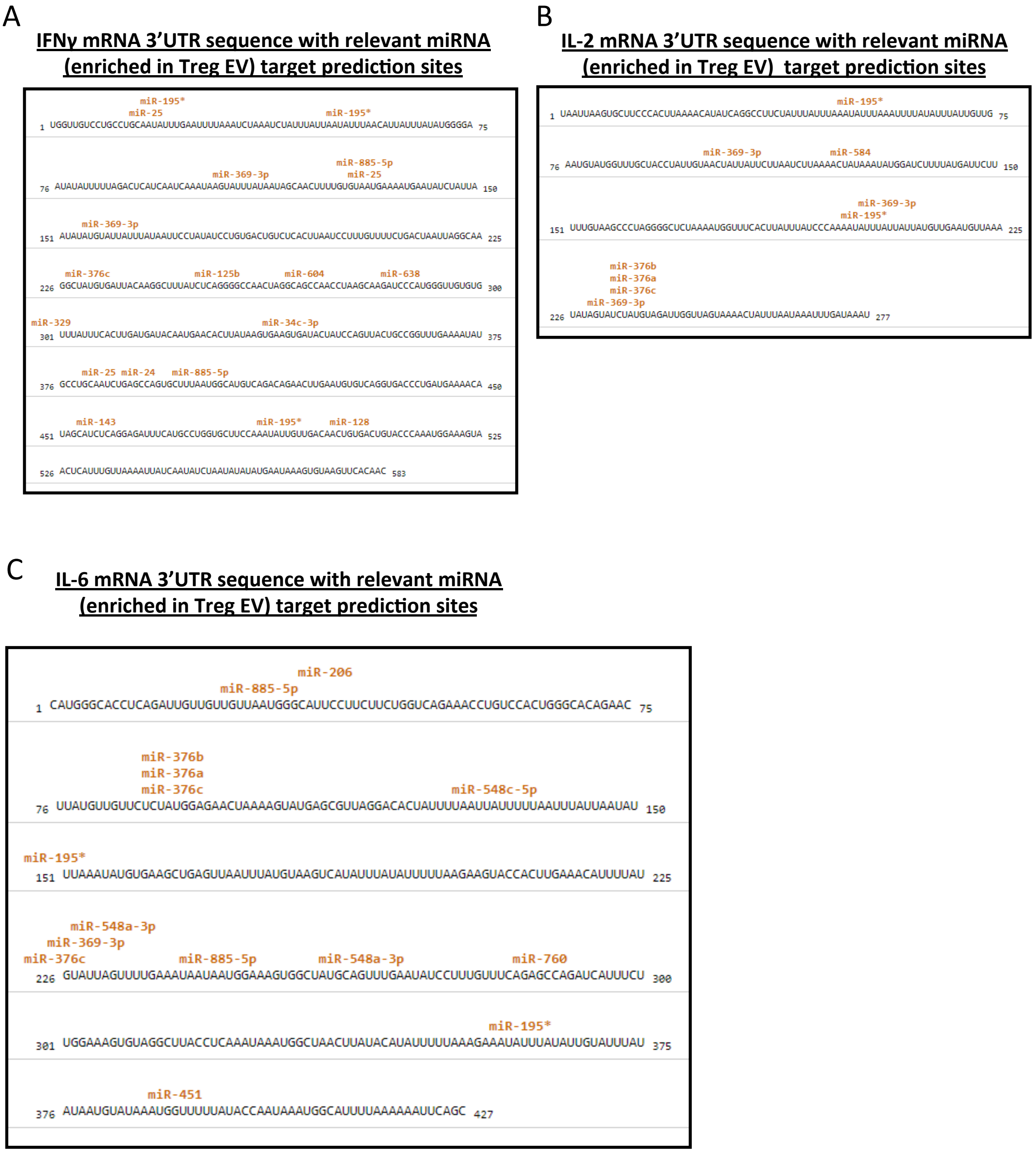
A
IFNγ mRNA 3’UTR sequence with relevant miRNA (enriched in Treg EV) target prediction sites
miR-195*
miR-25
miR-369-3p
miR-885-5p
miR-376c
miR-125b
miR-604
miR-638
miR-329
miR-34c-3p
miR-24
miR-143
miR-128
B
IL-2 mRNA 3’UTR sequence with relevant miRNA (enriched in Treg EV) target prediction sites
miR-195*
miR-369-3p
miR-584
miR-376b
miR-376a
miR-376c
C
IL-6 mRNA 3’UTR sequence with relevant miRNA (enriched in Treg EV) target prediction sites
miR-206
miR-885-5p
miR-376b
miR-376a
miR-376c
miR-548c-5p
miR-195*
miR-548a-3p
miR-369-3p
miR-760
miR-451

Fig. S3. Target prediction of miRNAs enriched in human Treg EVs. The bioinformatics tool microRNA.org was used to map the 3' UTR mRNA predicted targeting sites of (A) IFNγ (B) IL-2 and (C) IL-6 and to in silico predict their associated miRNA targets.

Figure S4.

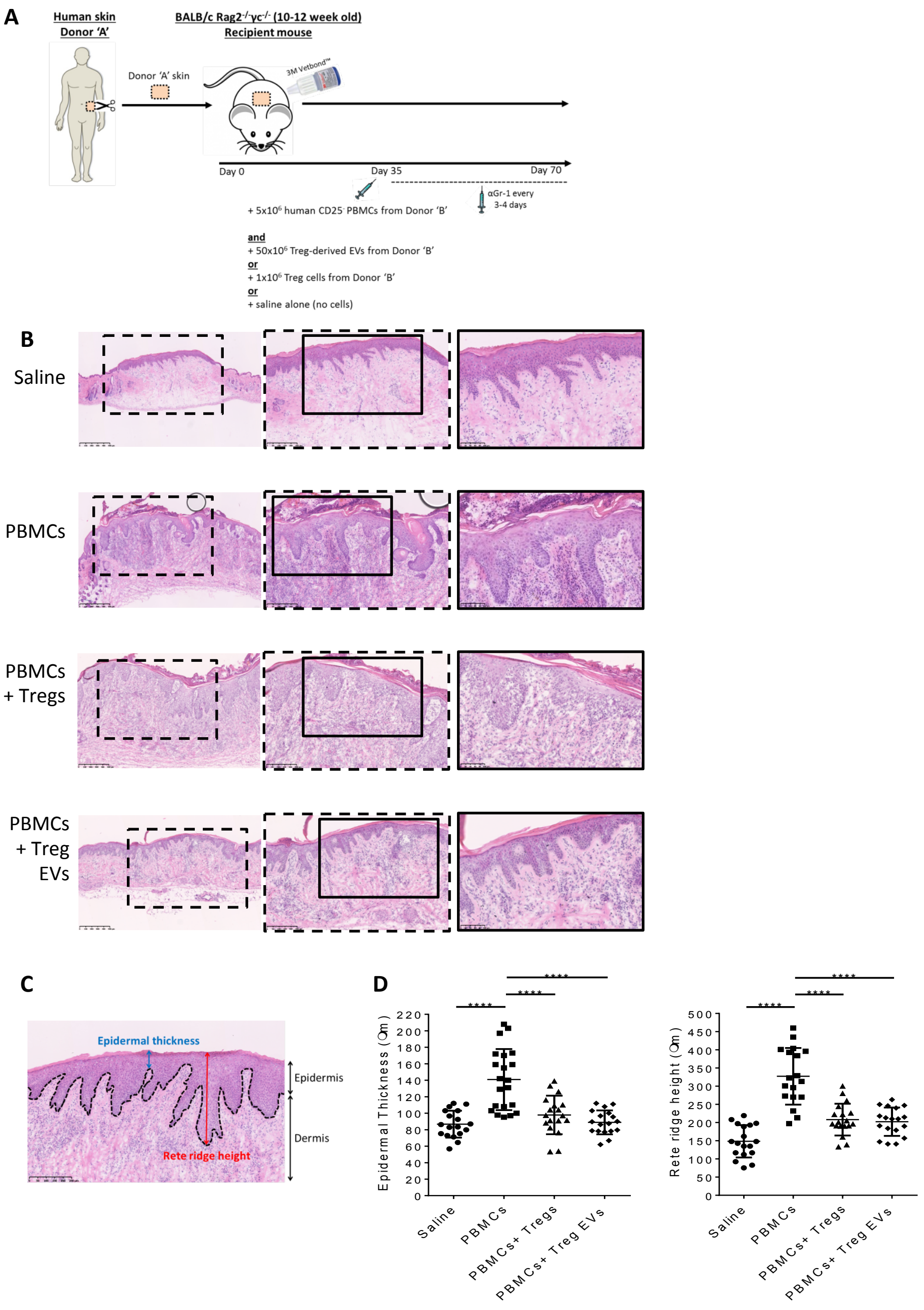

Fig. S4. H+E assessment of skin allograft injury following Treg EVs humanised mouse models of. (A)_Schematic diagram outlining the experimental procedure in BALB/c $Rag_2^{-/-}\gamma c^{-/-}$ mice of the human skin xenograft transplant and adoptive transfer of cells and EVs. **(B)** Skin allografts were stained with haematoxylin and eosin (H+E)

and images acquired using a high definition scanning light microscope. Representative images are shown. The left panel indicates a 5X objective with a scale bar of 500µm, the dotted box is enlarged and displayed in the middle panel which indicates a 10X objective with a scale bar of 250µm. The lined box is enlarged and displayed in the right panel and indicates a 20X objective with a scale bar of 100µm. (**C)** The epidermal and rete ridge heights measurement schematic, 5X objective. **(D)** Quantitation of both the epidermal (left panel) and rete ridge heights (right panel) were compared across the various treatments. Results represent 3-9 mice per group where four to six fields of view were quantified per section and data are representative of 5 individual experiments. Statistical significance was tested using one-way ANOVA and Turkey multiple comparison post-hoc test where *= $p<0.05$, **= $p<0.01$, ***= $p<0.001$, ****= $p<0.0001$ and ns= non-significant.

Figure S5.

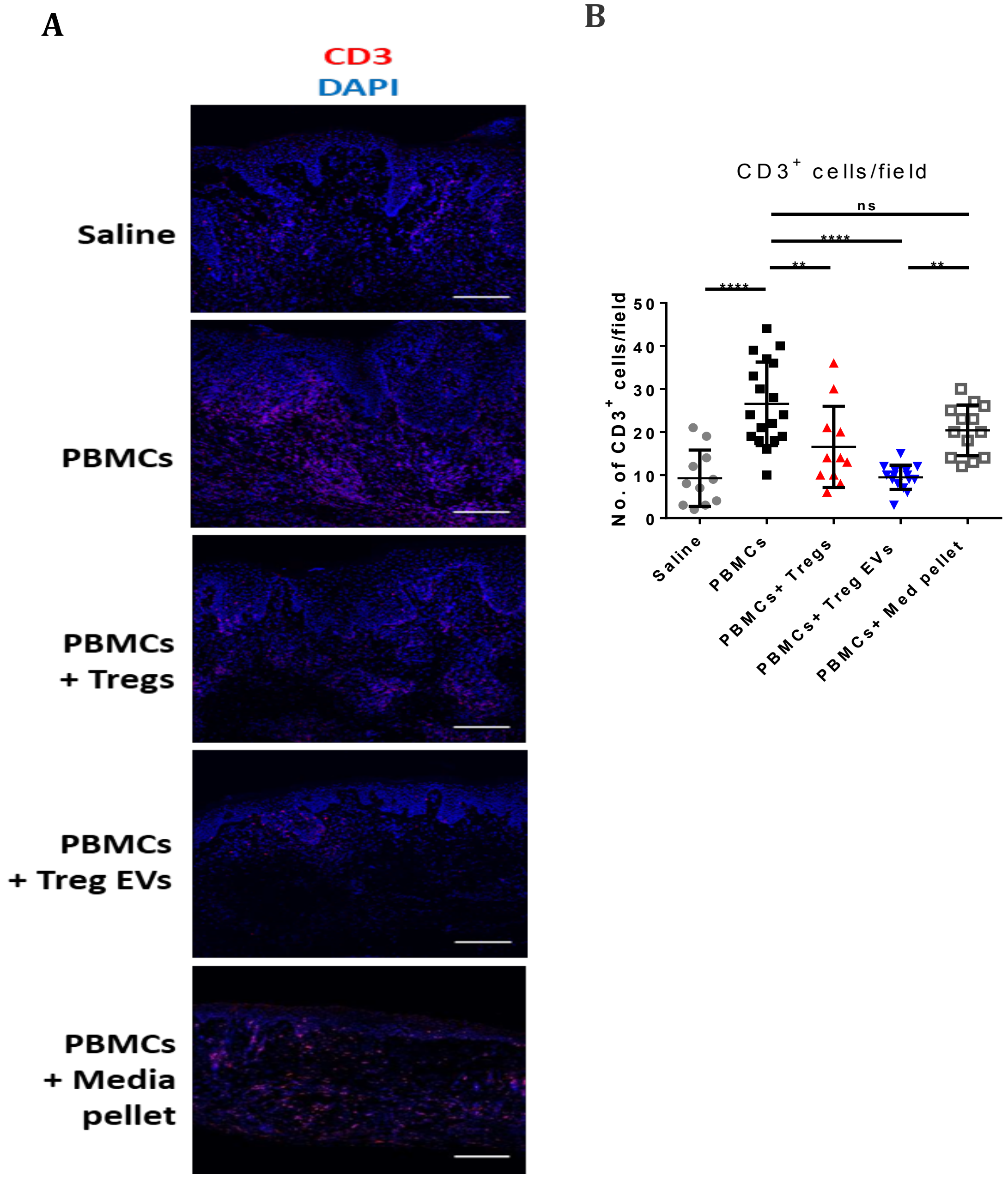

Fig. S5. Reduced CD3$^+$ T cells in humanised mouse models of skin allograft injury following Treg EV treatment. BALB/c Rag$^{-/-}\gamma^{-/-}$ mice were transplanted with human skin and BALB/c Rag2$^{-/-}\gamma c^{-/-}$ mice were transplanted with human skin and reconstituted with 5×10^6 allogenic CD4$^+$25$^-$ cells, at the same time some mice received either polyclonal 1×10^6 Tregs or Treg EVs derived from 50 x 10^6 Tregs.

Control mice received saline only or control EVs (media EVs). Human skin grafts were removed 5 weeks post-injection, cryopreserved sections were fixed and stained either for human CD3/DAPI **(A).** Images are representative two-colour immunofluorescence stains of human skin grafts. Representative images of human skin graft sections with the various treatments are shown. **(B)** Quantification of the number of human $CD3^+$ cells per field of view was performed using NIS Elements and FIJI imaging software. Results represent 3-9 mice per group where four to six fields of view were quantified per section and data are representative of 5 individual experiments. Statistical significance was tested using one-way ANOVA and Turkey multiple comparison post-hoc test where *= $p<0.05$, **= $p<0.01$, ***= $p<0.001$, ****= $p<0.0001$ and ns= non-significant.
